# Effects of continuous positive airway pressure on weight gain and metabolism: time for the muscles to step in!!!

**DOI:** 10.1093/sleep/zsaf302

**Published:** 2025-09-29

**Authors:** Akira Umeda, David Gozal

**Affiliations:** Department of General Medicine, School of Medicine, International University of Health and Welfare (IUHW), IUHW Shioya Hospital, Yaita-City, Tochigi-Prefecture, Japan; Office of the Dean and Departments of Pediatrics and Biomedical Sciences, Joan C. Edwards School of Medicine, Marshal University, 1600 Medical Center Drive, Huntington, WV, United States

In animal studies, chronic intermittent hypoxia (CIH) emulating the episodic desaturations of obstructive sleep apnea (OSA) is associated with either reduced or preserved somatic weight accrual and yet induces significant alterations in metabolism such as dyslipidemia and increased insulin resistance [1–4]. Furthermore, the role of adipose tissue dysfunction in the context of CIH has been recently challenged, whereby during the early stages of CIH the perturbation in oxygenation may actually enable some metabolic protection which then slowly disappears and is replaced by obvious metabolic dysfunction [3]. However, the effect of treatments mimicking continuous positive airway pressure (CPAP) conditions in murine models has not been specifically explored although one recent study showed some partial improvements in metabolic function [4].

In patients suffering from OSA, this issue has also not been explored in depth. For example, CPAP treatment appears to promote weight gain in patients with OSA, but the underlying mechanisms are still unclear [5, 6]. On the other hand, and somewhat paradoxically, adherence to CPAP and its accompanying weight gain were associated with a reduced risk of major adverse cardiac and cerebrovascular events recurrence [7]. In a study reported in this issue of SLEEP, Lee and colleagues report on their findings in an open label randomized controlled trial of OSA patients in which they compared changes in resting energy expenditure, energy/nutrition intake, physical activity, and body composition over 12 weeks among subjects randomly assigned to either CPAP therapy or control. Interestingly, they found that weight gain in CPAP-treated individuals did not appear to be due to changes in resting energy expenditure, physical activity, or nutritional intake; however, the authors postulate that weight gain was more likely related to changes in appetite-regulatory hormones and altered eating behaviors leading to some degree of positive energy balance [8]. Of note, the study was conducted during a transitional phase encompassing the COVID-19 pandemic, which may have imposed unrecognized challenges to the protocol being used. Notwithstanding, the cumulative findings point to the modest weight gain with CPAP originating from increases in fat-free mass (FFM) rather than from other sources (i.e. body fat).

How do these findings compare with those of other studies? Tachikawa et al. and Stenlöf et al. reported that energy expenditure during sleep significantly decreased after CPAP, but the former report described that dietary intake and eating behavior had greater impacts on weight change [9, 10]. Similarly, Shechter et al. and Münzer et al. performed before and after CPAP studies and similar to the current study, these investigators found that CPAP increased lean body mass in patients with OSA along with an overall increase in body weight [11, 12]. Since in normally hydrated healthy adults, the FFM and lean body mass differ only in the essential fat components [13], changes in FFM after CPAP can be interpreted as *de facto* changes in muscle mass. Therefore, increases in FFM or in muscle mass can be considered a favorable metabolic outcome in response to CPAP use.

In light of the putative effect of CPAP on FFM, does this imply that OSA causes muscle loss? Attention has tended to focus on the relationship between OSA and adiposity-related consequences, and the relationship between OSA and muscle function and structure has not received as much interest [14]. To this effect, Guo et al. reported that CIH (consisting of 2 min cycles alternating FiO_2_ 5%–6% 15 s and 21%, 8 h every day) significantly decreased the skeletal muscle index and cross-sectional area of the gastrocnemius muscle in Sprague–Dawley rats [15]. These investigators also reported that CIH resulted in the appearance of inward migration of muscle fiber cell nuclei and increased the distance between muscle cells, leading uneven cell size and shape, thereby suggesting the emergence of muscle atrophy. Similarly, Attaway et al. reported that CIH (i.e. 8 h hypoxia followed by 16 h normoxia) for three days led to global changes in protein synthesis and hypoxia inducible factor signaling using in vitro experiments mimicking the sarcopenia that occurs in patients with chronic obstructive pulmonary disease (COPD) [16]. They stated that this form of CIH may inhibit protein synthesis via dysregulation of hypoxia-inducible factors and lead to the emergence of sarcopenia. The analogy between these findings may be applicable to the FFM increases resulting from the initiation of CPAP in patients with OSA as reported by Lee and colleagues in this issue [8]. Indeed, reduced muscle protein synthesis, changes in muscle fiber distribution, insulin and lipid metabolism in muscle, and overall skeletal muscle mass may be altered in patients with OSA [14, 17–21]. All of these muscle derangements may be improved by CPAP and studies focusing on each of these elements are warranted.

Interestingly, Polat et al. reported that OSA patients had significantly greater muscle thickness and subcutaneous fat compared to controls using the data of thoracoabdominal CT scans [22]. Muscle thickness was positively associated with BMI and inversely associated with C-reactive protein levels and oxygen saturation. Although such findings may underlie increased fat deposition in the muscle rather than true hypertrophy, functional assessment in diverse muscles among patients with OSA are clearly necessary.

In summary, the effects of OSA on muscle and adipose tissues and the mechanisms governing such effects as well as their reversibility under CPAP treatment deserve future exploration. Lee et al. [8] have now added yet another important level of evidence in this direction.
